# Public knowledge, attitudes, and practices (KAP) regarding antibiotics use and antimicrobial resistance (AMR) in Bangladesh

**DOI:** 10.1016/j.heliyon.2023.e21166

**Published:** 2023-10-18

**Authors:** Md Ragaul Azim, K.M Nafiz Ifteakhar, Md Mahfujur Rahman, Quazi Nazmus Sakib

**Affiliations:** Institute of Health Economics, University of Dhaka, Dhaka, Bangladesh

**Keywords:** Antibiotics, Anti-microbial resistance, Public, KAP, Bangladesh

## Abstract

**Background:**

Antibiotic resistance is a global public health concern that requires an understanding of public knowledge, attitudes, and practices (KAP) towards antibiotics. This study aimed to assess the KAP regarding antibiotic use and AMR among the general population in Bangladesh.

**Methods:**

A cross-sectional survey was conducted among 656 respondents in Bangladesh. Data on socio-demographic characteristics, knowledge, attitudes, and practices regarding antibiotics use and AMR were collected through a structured questionnaire. Descriptive statistics and ordered logit regression analysis were performed to analyze the data.

**Results:**

The study involved participants, with 52.44 % aged 18 to 30, 77.29 % males, and 92.53 % having primary education or higher. Urban residents were 80 %, and students formed the largest occupational group (29.57 %), followed by businessmen (25 %), service holders (24.7 %), housewives (10.52 %), and day laborers (8.84 %). The majority of respondents demonstrated average knowledge (52.29 %), moderate attitudes (67.84 %), and good practices (50.61 %) regarding antibiotic use and AMR. Socioeconomic factors such as education, media exposure, and urban residence significantly (1 % level of significance) influenced KAP. The findings revealed knowledge gaps and misconceptions among the respondents, including incorrect beliefs about antibiotic resistance (42%), and inadequate awareness of the importance of completing full antibiotic courses (54.88 %).

**Conclusion:**

This study sheds light on the existing KAP related to antibiotic use and AMR within the general population of Bangladesh. The findings reveal varying levels of knowledge, attitudes, and practices among the participants. The results underscore the importance of addressing knowledge gaps, and misconceptions for awareness building through educational campaigns utilizing social media platforms and newspapers. These insights provide a foundation for informed strategies to ensure the continued efficacy of antibiotics.

## 1 Introduction

Antibiotic and antimicrobial resistance (AMR) have become a significant global health concern, posing a threat to the effective treatment of infectious diseases [1]. AMR jeopardizes the effectiveness of antibiotics. The emergence and spread of drug-resistant pathogens have led to increased morbidity, mortality, and healthcare costs worldwide [2]. A report from the Organization for Economic Co-operation and Development (OECD) in 2018 predicted that infections with resistant microorganisms will result in 2.4 million deaths over the next 30 years in Europe, North America, and Australia and cost up to US$3.5 billion annually across 33 OECD and EU countries [3]. Globally, an estimated 4.95 million deaths related to bacterial antimicrobial resistance (AMR) occurred in 2019, with 1.27 million of those deaths directly attributable to bacterial AMR alone [4]. The situation is particularly critical in low- and middle-income countries, where factors such as limited resources, inadequate healthcare infrastructure, and poor antibiotic stewardship exacerbate the problem [5]. Risk assessments conducted by the World Health Organization (WHO) find that the South-East Asia region, including Bangladesh, to be the most at-risk part of the world for AMR [6]. A country-wide AMR surveillance in Bangladesh reveals an alarming situation, as many bacteria including Escherichia coli, Klebsiella pneumoniae, Staphylococcus aureus, Salmonella spp, and Acb complex are already resistant to commonly used antibiotics as well as some reserve groups [7]. Many of the pathogens also exhibit significant resistance to commonly employed antibiotics in countries such as Pakistan, India, Sri Lanka, and the Philippines [[8], [9], [10], [11], [12]].

Globally, the misuse and overuse of antibiotics are two major causes contributing to the development of antibiotic-resistant pathogens [13]. Bangladesh, as a densely populated country with a high burden of infectious diseases, faces substantial challenges in managing AMR [14]. All the main factors contributing to the emergence of AMR in Bangladesh can be categorized into the supply and demand sides. On the supply side, factors include the excessive and inappropriate prescribing of antibiotics by practitioners, lack of diagnostic facilities, and aggressive marketing strategies of pharmaceutical companies. On the demand side, the primary drivers behind increasing antimicrobial use consist of poor consumer awareness of the dangers of inappropriate use, non-compliance with dosage, and self-medication [15]. Multiple studies have reported that a large number of consumers in Bangladesh take antimicrobials without a licensed medical doctor's prescription. Therefore, poor consumer awareness has proven to be the most vital factor contributing to the development of AMR [16,17].

A large portion of the population's inadequate knowledge, attitudes, and practices regarding antibiotic use contribute to misuse and inappropriate utilization [18]. Understanding the knowledge, attitudes, and practices (KAP) of the general public regarding antibiotics and AMR is essential for developing targeted interventions and promoting responsible antibiotic use. Despite the global concern over AMR, limited attention has been given to understanding the current conceptions and practices related to antibiotics among the general population in Bangladesh. The existing body of literature predominantly concentrated on assessing KAP within distinct demographic segments in Bangladesh, encompassing healthcare providers, urban populations, poultry farmers, and specific student cohorts like undergraduate pharmacy students, veterinary students, and public university students [[19], [20], [21], [22], [23], [24]]. Various KAP-based studies also conducted across different nations of the world to understand the KAP of the general people [[25], [26], [27], [28]]. However, a notable gap persists in assessing KAP pertaining to the general populace in Bangladesh.

This study aimed to investigate the general public's KAP regarding antibiotics use and AMR in Bangladesh. By conducting a comprehensive study on the KAP of the general population, valuable insights can be obtained to develop targeted awareness campaigns, educational initiatives, and policy interventions. This research would provide a baseline understanding of the current situation and help identifying specific areas where interventions are needed to address knowledge gaps, dispel misconceptions, and promote responsible antibiotic use among the general population. The findings of the study will also fill the existing knowledge gap and contribute to global efforts in combating AMR. It will enable healthcare authorities, policymakers, and public health professionals to design evidence-based interventions tailored to the specific needs and challenges faced by the general population in Bangladesh to reduce the burden of AMR, and improve overall healthcare outcomes. Successfully combating antimicrobial resistance is closely associated with achieving 6 out of the 17 Sustainable Development Goals (SDGs), in particular, SDG 3 [29].

### 1.1 A brief definition of KAP regarding antibiotics use and AMR

Knowledge, Attitudes, and Practices (KAP) regarding antibiotics use and Antimicrobial Resistance (AMR) refer to a comprehensive assessment of individuals' understanding, beliefs, and behaviors related to the use of antibiotics and the development of antibiotic resistance. Knowledge encompasses the level of awareness and information individuals possess about antibiotics, their appropriate use, and the consequences of antibiotic resistance. Attitudes encompass individuals' perceptions, beliefs, and attitudes towards antibiotics, including their perceptions of antibiotic effectiveness, risks, and benefits. Practices involve the actual behaviors and actions individuals engage in when using antibiotics, encompassing adherence to prescriptions, self-medication patterns, and other related behaviors.

## 2 Methods and materials

### 2.1 Sampling methods

The study employed a cross-sectional study design and utilized a quantitative method to analyze primary data collected from December 2022 to January 2023. Data collection took place at five major public places where people gather in Dhaka city, namely marketplaces, railway stations, bus stations, educational institutions, and launch terminals. The objective was to assess the knowledge, attitudes, and practices (KAP) of the general population regarding antibiotics use and AMR in Bangladesh. The selection of these specific public places was based on multiple reasons, including the fact that they serve as the country's frequent visited entry and exit points by the people from all socio-economic background traveling within the country. These locations attract a diverse cross-section of the general population. By selecting these places, we can capture the perspectives and behaviors of the local population and individuals visiting or passing through Dhaka from other parts of Bangladesh. This provides us with a unique opportunity to assess the knowledge, attitudes, and practices regarding antibiotic use and AMR among diverse individuals, including residents, travelers, and individuals from different socio-cultural backgrounds.

To determine the appropriate sample size, the study utilized the single population proportion formula with a 95 % confidence interval, assuming a 50 % prevalence, 5 % margin of error, and a design effect of 1.7. Based on these parameters, the required sample size is 653. This ensures a 95 % confidence level that the true value falls within ±5 % of the estimated value. As the sampling design does not follow a systematic random sampling technique, the study chose a design effect of 1.7. As we conduct the survey in the public places, a higher non-response rate of 10 % was being anticipated. Hence, the targeted sample size was set at 718. However, during the data collection phase, the study accounted the actual non-response rate is 9.05 %. As a result, the final sample size of the study were 656 respondents, which are larger than the required sample size. The sampling method used was convenience sampling, which is deemed appropriate given the nature of data collection in public places. The study employed an exclusion criterion of not interviewing individuals below 18 years of age to ensure the ethical and legal requirements were met. By employing this study design and data collection approach, the research aims to gather insights into the KAP of the general population.

### 2.2 Data collection instruments (DCIs)

To conduct the survey, a structured questionnaire was utilized, comprising the socio-demographic characteristics of the respondents and various questions to assess their knowledge, attitudes, and practices concerning antibiotic use and antimicrobial resistance (AMR). The questionnaire was developed based on a thorough literature review of similar studies conducted in different settings, with necessary modifications to align with the context of Bangladesh [20,22,23,25,26,30]. The questionnaire encompassed four main sections [1]: socio-demographic characteristics of the participants, such as gender, age, place of residence, marital status, level of education, occupation, monthly income, monthly family income, and media exposure [2]; knowledge about antibiotic use and AMR, comprising eight questions [3]; attitudes toward antibiotic use and AMR, consisting of eight questions; and [4] self-reported practices related to antibiotic use, comprising 12 questions. The first four questions in this section directly assessed overall practice behavior, while the remaining eight focused on practice behavior within the past 12 months, if applicable. The respondents' knowledge regarding antibiotic use and AMR was assessed using “yes, no, do not know” responses, while their attitudes were measured through “agree, disagree, neutral” responses. Practices related to antibiotic were captured using nominal or categorical (yes or no) responses. For instance, the frequency of antibiotic intake was gauged using five nominal responses, including “with physician's prescription, self-medication, suggested by friends or relatives, suggested by medicine seller, following the previous prescription.” Furthermore, the reason for taking the last antibiotic was categorized into responses such as “cold, fever, bacterial infection, others."

A psychometric analysis was conducted to evaluate the construct validity and reliability of the survey tools employed in this study. Construct validity was assessed using factor analysis and Cronbach's alpha. Confirmatory Factor Analysis (CFA) was employed to estimate factor loadings, which represent the correlation between responses to the survey questions and the unobserved construct (e.g., knowledge, attitudes, and practices). The factor analysis results supported the questionnaire's construct validity, as indicated by factor loadings surpassing the recommended threshold of 0.40 or 0.50. To evaluate internal consistency reliability, Cronbach's alpha coefficient was calculated for each questionnaire scale. Cronbach's alpha values assess how well the items within a scale align with each other to measure the same underlying construct. In our study, Cronbach's alpha values ranged from 0.5 to 0.6 for the different scales, indicating high internal consistency. Moreover, a pilot study was conducted to assess the clarity, relevance, and comprehensibility of the questionnaire items. Revisions were made based on the pilot results. The questionnaire was then translated into Bengali, the native language spoken by the study participants, to ensure accessibility and cultural appropriateness. Trained enumerators administered the questionnaire to the participants. Overall, the steps taken to ensure the reliability and validity of the questionnaire, including the assessment of internal consistency, content validity, construct validity, and pilot testing, contribute to the robustness and credibility of the data collected in this study. Ethical approval (IHEirb562022) for the study was obtained from the Institutional Review Board (IRB) of the Institute of Health Economics, University of Dhaka. Verbal informed consent was obtained from all participants after comprehensively explaining the study's purpose, nature, and voluntary participation. Participants were assured of the confidentiality of their responses and their right to withdraw from the study at any time without facing any consequences. Verbal consent was documented by the research team and notarized by an impartial witness present during the consent process.

### 2.3 Data analysis

The collected data underwent descriptive statistics, bivariate analysis, and multivariate analysis. Descriptive statistics were employed to calculate the percentage of each response across all participants for the knowledge, attitudes, and practices sections. Specifically, in the knowledge questions, responses of “yes” to indicators 2 and 7 in Table 2 were categorized as “incorrect knowledge,” while all other parameters with “yes” responses were considered “correct knowledge.” Similarly, in the attitude questions, a response of “disagree” to parameter 2 in attitude section of Table 2 was classified as a “negative attitude,” while the remaining parameters with “disagree” responses were labeled as “positive attitude.” Regarding the practice questions, seven out of the twelve questions focused on respondents' actual behavior, while the remaining questions served as connecting points. Among these seven questions, a “yes” response to parameters 1, 2, and 4 in Table 3 was considered “good practice.” Additionally, parameter 3 was deemed “good practice” only when the response indicated "physician's prescription".

**Table 1:** Principal characteristics of the study participants.

Variables	Percentage (%)	n (N = 656)
Age		
18 - <30	52.44	344
30 - <50	37.2	244
50 - <60	7.93	52
=>60	2.44	16
Gender		
Male	77.29	507
Female	22.71	149
Monthly Family Income		
Low (<20000BDT)	21.65	142
Middle (>=20000 <=50000BDT)	64.02	420
High (>=50000 BDT)	14.33	94
Education		
Less than primary	7.47	49
Primary passed	14.63	96
SSC passed	17.23	113
HSC passed	38.41	252
Graduation and above	22.26	146
Marital Status		
Married	58.54	384
Unmarried	41.46	272
Place of Residence		
Urban	80.34	527
Rural	19.66	129
Occupation		
Business	25.0	164
Day labor	8.84	58
Service	24.7	162
Housewife	10.52	69
Student	29.57	194
Others	1.37	9
Main Media Exposure		
TV	29.73	195
Newspaper	7.93	52
Radio	0.76	346
Social media	52.74	5
No media exposure	8.84	58
Household Head Occupation		
Business	42.84	281
Day labor	10.67	70
Service	41.62	273
Housewife	0.3	2
Other	4.57	30
Knowledge Score		
Low (<=40)	36.89	242
Medium (>40 - <80)	52.29	343
High (=>80)	10.82	71
Attitude Score		
Negative (<=40)	20.12	132
Moderate (>40 - <80)	67.84	445
Positive (=>80)	12.04	79
Practice Score		
Poor (<=40)	19.36	127
Medium (>40 - <80)	30.03	197
Good (=>80)	50.61	332

-SSC: Secondary School Certificate

-HSC: Higher Secondary Certificate

-TV: Television

**Table 2:** Status of knowledge and attitudes regarding antibiotics use and AMR of the respondents.

	Knowledge indicators N = 656	Correct knowledge % (n)	Incorrect knowledge % (n)
1	Antibiotics are effective for the treatment of bacterial infections	80.49 (528)	19.51 (128)
2	Antibiotics are effective for the treatment of viral infections	9.3 (61)	90.7 (595)
3	Loss of effectiveness of an antibiotic is antibiotic resistance	43.75 (287)	56.25 (369)
4	Antibiotic resistance creases due to missing of an antibiotic dose	71.95 (472)	28.05 (184)
5	The more we use antibiotic, the higher the risk that resistance develops	58.38 (383)	41.62 (273)
6	Antibiotic resistance can develop due to use of antibiotic without doctor's prescription	44.82 (294)	55.18 (362)
7	After feeling better, one should stop a partially completed antibiotic dose	45.12 (296)	54.88 (360)
8	Antibiotic resistance can develop due to misuse of antibiotic	58.08 (381)	41.92 (275)
	**Attitude Indicators**	**Correct attitude** **% (n)**	**Incorrect attitude** **% (n)**
1	Antibiotics are safe, so they can commonly be used	69.66 (457)	30.34 (199)
2	Without prescription, sale of antibiotics should be banned	74.09 (486)	25.91 (170)
3	Surplus/unused antibiotics can be saved for future use or to give someone else	72.1 (473)	27.9 (183)
4	Antibiotics speed up the recovery from most coughs and colds	15.24 (100)	84.76 (556)
5	It is okay to take antibiotics based on the suggestion of a medicine seller	77.9 (511)	22.1 (145)
6	I should take antibiotics to avoid getting a more severe illness, when I suffer from cough and cold	44.82 (294)	55.18 (362)
7	Antibiotic speeds up recovery when I get a fever	16.77 (110)	83.23 (546)
8	If a doctor does not prescribe an antibiotic when I think one is needed, I will go to another doctor	9.91 (65)	90.09 (591)

- AMR: Antimicrobial Resistance.

**Table 3:** Status of practices regarding antibiotics and AMR of the respondents.

	Practice Indicators	Response	%	n (N = 656)
1	Whether doctor used to be consulted before using antibiotic	Yes	82.93	544
		No	17.07	112
2	Did the antibiotic course is fulfilled?	Yes	60.98	400
		No	39.02	256
3	How did the antibiotics is taken?	Physician's prescription	77.9	511
		Self-medication	3.2	21
		Suggested by friends/relatives	0.91	6
		Suggested by medicine seller	16.31	107
		According to previous prescription	1.68	11
4	If feel better after taking some doses of antibiotics, did the dose still be completed	Yes	63.26	415
		No	36.74	241
The fulfillment status of the last course of antibiotics based on the person suggesting the use of antibiotics, according to respondent's latest intake (n = 358)^a^				
	**Parameters**	**Actions**	**%**	**n**
	Physician's prescription (n = 281)	Course completed	80.43	226
		Course not completed	19.57	55
	Self-medication (n = 19)	Course completed	21.05	4
		Course not completed	78.95	15
	Suggested by friends/relatives (n = 4)	Course completed	75.00	3
		Course not completed	25.00	1
	Suggested by medicine seller (n = 44)	Course completed	47.73	21
		Course not completed	52.27	23
	According to previous prescription (n = 10)	Course completed	60.00	6
		Course not completed	40.00	4

- AMR: Antimicrobial Resistance.

a A total of 358 respondents reported antibiotic uptake among the 656 participants, during the last 12 months from the survey period. This response pertains to those respondents who had utilized antibiotics within the preceding 12-month timeframe.

A scoring system was implemented to measure respondents' knowledge, attitudes, and practices toward antibiotic. Each correct response (indicating correct knowledge, positive attitude, or good practice) received a value of 1, while incorrect responses (incorrect knowledge, do not know, negative attitude, neutral, and poor practice) received a value of 0. Participants' overall knowledge, attitudes, and practices were then categorized using the modified Bloom's cut-off point. A score between 80 and 100 % (7–8 points) was classified as high/positive/good, a score ranging from 41 to 79 % (4–6 points) as medium/moderate, and a score of 40 % or less (0–3 points) as low/negative/poor. This categorization helped assess the study population's knowledge, attitudes, and practices.

We employed ordered logit regression models to explore potential determinants of KAP regarding antibiotic use and AMR. The ordered logit regression was chosen as the dependent variables showing whether an individual has high, moderate or low level of KAPs which is ordinal in nature. Hence, to analyze the relationships between the ordinal dependent variables (KAP scores) and the explanatory variables, order logit model could provide insights into the important determinants of knowledge, attitudes, and practices related to antibiotic use and AMR within the study population. We ran five distinct regression models to examine the relationships between the KAP variables and various explanatory factors. Model 1 utilized the knowledge level as the dependent variable, while Model 2 and Model 3, the attitude level and practice level served as the dependent variables respectively. In all three cases different socio-economic variables (i.e., age, education, income, gender, marital status, place of residence, occupation, and household head occupation) and main media exposure were considered as explanatory variables. Model 4 is same as Model 2 except, in addition to the earlier explanatory variables, knowledge level was included in the model as an explanatory variable. This was done based on the assumption that attitude may be affected by the level of knowledge. Similarly, based on the assumption that knowledge and attitude levels can affect practice, Model 5 attempted to explain level of practice having knowledge and attitude level as explanatory variables in addition to the ones used in Model 3. The results of these regression models were analyzed by examining the average marginal effects of each explanatory variable. The robust standard error, presented in parentheses in the table, indicates the level of precision in the estimation.

## 3 Results

### 3.1 Background characteristics

The study included 656 respondents, with 52.44 % falling within the age range of 18–30 years. Males constituted 77.29 % of the participants. The majority (64.02 %) belonged to middle-class families with a monthly income ranging from BDT 20,000 to 50,000. This suggests that the study encompassed individuals from a diverse range of income backgrounds. A significant majority (92.53 %) had at least a primary education, with the largest educational group (38.41 %) having completed higher secondary education, followed by the “Graduation and above” group (22.26 %). Marital status data indicated that approximately 58 % of the respondents were married. Almost four-fifths of the respondents were urban residents, indicating a higher representation from urban areas compared to rural areas. Occupation-wise distribution revealed that the largest proportion of respondents, comprising 29.57 %, were students. Other significant groups included businessmen (25 %), service holders (24.7 %), housewives (10.52 %), and day laborers (8.84 %). This indicates a diverse mix of occupational backgrounds among the study participants. Social media was the primary source of information for more than half of the respondents, followed by TV (29.73 %), newspapers (7.73 %), and radio (0.76 %). Notably, 8.84 % reported no exposure to any media. The majority of household heads were businessmen (42.84 %) or service holders (41.62 %). Detailed socio-demographic characteristics are provided in Table 1.

## 4 Public knowledge regarding antibiotics use and AMR

The majority of respondents (52.29 %) demonstrated average knowledge, while a small portion (10.82 %) exhibited high knowledge (Table 1). Approximately 80 % of respondents were aware of antibiotics' efficacy in treating bacterial infections, and nearly 72 % recognized the connection between missed antibiotic doses and the development of antibiotic resistance (Table 2). However, around 42 % of respondents possessed incorrect beliefs about antibiotic resistance stemming from misuse, and a similar percentage lacked awareness that increased antibiotic usage could heighten resistance risks. Notably, over 90 % of respondents were unaware that antibiotics are ineffective against viral infections. Furthermore, more than half (56 %) of the participants were unfamiliar with the concept that antibiotic resistance signifies a decline in antibiotic effectiveness, and a comparable number failed to acknowledge that using antibiotics without a doctor's prescription can contribute to resistance (Table 2). Additionally, over half of the respondents lacked knowledge regarding the importance of completing a full antibiotic course, even if symptoms improve.

### 4.1 Public attitude regarding antibiotics use and AMR

The majority of respondents (67.84 %) demonstrated a moderate attitude towards antibiotic uses and AMR, while the percentage of respondents with a positive attitude (12.04 %) was notably lower than those with a negative attitude (20.12 %) (Table 1). A significant proportion (77.9 %) of respondents correctly agreed that taking antibiotics based on the suggestion of a drug seller is unacceptable. Furthermore, a substantial majority (74.09 %) concurred that the sale of antibiotics without a prescription should be prohibited, and approximately 72 % of respondents believed that surplus or unused antibiotics should not be stored for future use or given to someone else. Conversely, a notable proportion of respondents (83.23 %) held the misconception that antibiotics can hasten the recovery process after experiencing fever. Likewise, a similar percentage of respondents (81.1 %) agreed that if they believed antibiotics were necessary, they should seek treatment from another doctor if the initial doctor did not prescribe them (Table 2).

### 4.2 Public practice regarding antibiotics and AMR

In terms of practice, approximately half of the respondents (50.61 %) exhibited good practices, while 19.36 % demonstrated poor practices regarding antibiotic use (Table 1). Nearly 83 % of respondents sought a physician's consent before using antibiotics. Around 61 % of respondents consistently completed their antibiotic course, and approximately 77.9 % of them only took antibiotics if prescribed by a physician. Furthermore, 63.26 % of respondents completed their course even if they felt better after taking some doses (Table 3). Based on the respondents' experiences from their last course of antibiotics, it is evident that over 80 % of respondents completed their antibiotic course when it was prescribed by a physician. However, only 21 % of respondents adhered to this guideline when they self-medicated with antibiotics. Additionally, when examining respondents' antibiotic intake patterns for different diseases based on their most recent experiences, it is notable that the highest tendency to seek a physician's advice before taking antibiotics was observed for cold/dust allergy (100 %), sinusitis (87.50 %), and urinary tract infection (80.00 %), respectively. In contrast, the highest tendency to take antibiotics without any doctor's advice was observed for ulcers (28.57 %), followed by heart disease (25 %).

### 4.3 Socio-economic determinants affecting KAP about antibiotics use and AMR

Among the socio-economic factors influencing individuals' knowledge, attitudes, and practices regarding antibiotic use and AMR, education appears to have the most notable effect. Respondents with a graduation or above education demonstrated higher levels of knowledge (19.18 %), positive attitudes (22.60 %), and good practices (70.55 %) compared to other education groups (Table 4). Similarly, respondents with HSC-level education exhibited better knowledge (12.70 %), attitudes (11.51 %), and practices (56.35 %) than those with primary, less than primary, and SSC-level education.

**Table 4:** Level of knowledge, attitudes, and practices regarding antibiotics use and AMR of the respondents based on their socio-economic conditions.

Variables	Knowledge			Attitudes			Practices		
	Low ( ≤ 40)	Medium (>40-<80)	High ( ≥ 80)	Negative ( ≤ 40)	Moderate (>40-<80)	Positive ( ≥ 80)	Poor ( ≤ 40)	Medium (>40-<80)	Good ( ≥ 80)
Gender									
Male	36.91	52.35	10.74	20.13	70.47	9.40	10.07	34.23	55.70
Female	36.88	52.27	10.85	20.12	67.06	12.82	22.09	28.80	49.11
Education									
Less than primary	75.51	24.49	0.00	63.27	32.65	4.08	61.22	16.33	22.45
Primary	63.54	33.33	3.13	18.75	75.00	6.25	42.71	33.33	23.96
SSC	45.13	47.79	7.08	21.24	70.80	7.96	14.16	38.94	46.90
HSC	28.17	59.13	12.70	16.27	72.22	11.51	10.71	32.94	56.35
Graduation and above	15.07	65.75	19.18	12.33	65.07	22.60	8.90	20.55	70.55
Media Exposure									
TV only	46.15	47.69	6.15	24.10	68.21	7.69	14.36	33.33	52.31
Newspaper	11.54	73.08	15.38	11.54	71.15	17.31	5.77	30.77	63.46
Social media	30.06	55.78	14.16	15.90	69.65	14.45	17.92	30.06	52.02
Radio	0.00	100.00	0.00	0.00	60.00	40.00	20.00	20.00	60.00
No media exposure	72.41	24.14	3.45	41.38	53.45	5.17	56.90	18.97	24.14
Place of Residence									
Urban	33.97	54.46	11.57	18.22	69.26	12.52	18.60	26.76	54.65
Rural	48.84	43.41	7.75	27.91	62.02	10.08	22.48	43.41	34.11
Age									
Age 18-<30	29.07	57.85	13.08	16.86	71.51	11.63	16.28	31.98	51.74
Age 30-<50	44.26	46.31	9.43	22.13	64.34	13.52	23.36	26.23	50.41
Age 50-<60	51.92	44.23	3.85	34.62	57.69	7.69	23.08	34.62	42.31
Age=>60	43.75	50.00	6.25	12.50	75.00	12.50	12.50	31.25	56.25

-SSC: Secondary School Certificate

-HSC: Higher Secondary Certificate

-TV: Television

-AMR: Antimicrobial Resistance

Likewise, media exposure plays a significant role in KAP, with respondents having no media exposure showing lower levels of knowledge (3.45 %), poorer attitudes (5.17 %), and worse practices (24.14 %) compared to those with any form of media exposure (Table 4). Specifically, access to newspapers had a more pronounced positive impact on knowledge (15.38 %) and practice levels (63.46 %) compared to other forms of media. Furthermore, access to social media had a greater influence on attitudes (40 %) than other types of media. Respondents residing in urban areas displayed higher scores in terms of knowledge (11.57 %), attitudes (12.52 %), and practices (54.65 %) compared to their rural counterparts. The age group of 18–30 years exhibited better knowledge (13.08 %), the age group of 30–50 years showed better attitudes (13.52 %), and respondents above 60 years old displayed better practices (56.25 %) than other age groups. Conversely, respondents' gender had no significant impact on their knowledge, attitudes, and practices levels.

## 5 Results of ordered logit regression

Education emerged as a significant determinant of KAPs, demonstrating statistical significance at a 1 % level across all five models (Table 5). Income, measured in thousands of BDT, showed significance only in the regression of knowledge and practice, although the effect size was negligible. However, when controlling for knowledge and attitude scores, income became insignificant (at a 10 % level of significance) in the regression of practice. The male dummy variable exhibited significance in the regression of attitude, indicating that males have a 5 % higher probability of having a high level of attitude. However, after controlling for knowledge score, the male dummy variable became insignificant. The urban dummy variable was significant at a 1 % level of significance in the regression of knowledge and practice when knowledge and attitude were included in the regression (Model 5), suggesting that residing in an urban area positively affects knowledge and practice. Among the occupation dummies, only the service dummy variable was significant in explaining KAPs, indicating that service holders have a greater probability of having a high level of knowledge, attitude, and practice compared to day laborers. However, after controlling for knowledge level, the service dummy variable had no significant effect on attitude. Similarly, housewives exhibited a higher probability of having a high level of attitude and practice, but no significant relationship was found between knowledge level and being a housewife.

**Table 5:** Regression models for potential determinants of the KAP regarding antibiotics use and AMR using ordered logit.

Variables	Model 1 (Dependent variable is knowledge score	Model 2 (Dependent variable is attitude score)	Model 3 (Dependent variable is practice score)	Model 4 (Dependent variable is attitude score)	Model 5 (Dependent variable is practice score)
Knowledge score	–	–	–	0.093*** (0.015)	0.104*** (0.03)
Attitude score	–	–	–	–	0.057* (0.034)
Practice score	–	–	–	–	–
Age	−.00085 (0.00085)	0.0003 (.0009)	0.0034* (.002)	0.00050 (0.00099)	0.0032* (0.002)
Education	0.013*** (0.0027)	0.012*** (.003)	0.023*** (0.005)	0.0086*** (0.002)	0.017248*** 0.005653
Monthly income (in 000 BDT)	0.00047* (0.00026)	0.000343 (0.00025)	0.0020* (0.0013)	0.0002 (0.0002)	0.00156 0.00136
Male dummy (=1 if male, base category is female)	0.0258 (0.023)	0.059* (0.033)	−0.028 (0.05)	0.048 (0.031)	−0.045 (0.056)
Married dummy (=1 if married, base category unmarries)	−0.0047 (.022)	0.037 (0.028)	0.065 (0.052)	0.04 (0.03)	0.068 (0.051)
Urban dummy	0.0178* (.0203)	0.0133 (0.023)	0.077 (.039)	0.009 (0.020)	0.077* (0.038)
Occupation (Base category is day labor)					
Business Dummy (=1 if occupation is business)	−0.012 (0.043)	0.0046 (0.041)	0.016* (0.091)	0.011 (0.041)	0.157* (0.088)
Student Dummy (=1 if student)	0.03 (0.045)	0.03 (0.045)	0.29*** (0.097)	0.024 (0.044)	0.272*** (0.092)
Service Dummy (=1 if occupation is service)	0.07* (0.043)	0.077* (0.042)	0.26*** (0.095)	0.061 (0.042)	0.221** (0.090)
Housewife Dummy (=1 if housewife)	0.034 (0.050)	0.094** (0.048)	0.183* (0.10)	0.087* (0.047)	0.159* (0.097)
Media Exposure (Base category no media exposure)					
TV exposure dummy (=1 if TV is main media exposure)	0.026 (0.033)	0.009 (0.033)	0.16* (0.074)	0.008 (0.030)	0.155** (0.070)
Newspaper exposure dummy (=1 if newspaper is main media exposure)	0.095** (0.039)	0.059* (0.044)	0.15* (.093)	0.036 (0.043)	0.103 (0.091)
Radio dummy (=1 if radio is main media exposure)	0.17* (0.068)	0.287*** (.078)	0.48*** (0.156)	0.247*** (0.077)	0.359** (0.154)
Social media dummy (=1 if social media is main media exposure)	0.045 (0.035)	0.059 (0.035)	0.10 (0.078)	0.050 (0.033)	0.078 (0.074)
Household Occupation (base category day labor)					
HH Business (=1 if household head occupation is business)	0.027 (0.034)	−0.007 (0.033)	0.092 (0.073)	−0.017 (0.033)	0.090 (0.069)
HH service (=1 if household head occupation is service)	0.0015 (0.034)	−0.020 (0.032)	0.118*** (0.072)	−0.021 (0.0321)	0.126* (0.069)
n	**656**	**656**	**656**	**656**	**656**

-The result shows the average marginal effects of each of the explanatory variables

-The number in the parenthesis represent the robust standard error

-‘***’- significant at 1 % level of significance, ‘**’- significant at 5 % level of significance, ‘*’ significant at 10 % level of significance

-KAP: Knowledge, attitudes, and practices

-HH: Household Head

Individuals who primarily relied on newspapers as their source of media exposure were more likely to have a higher probability of possessing knowledge, attitude, and practice. However, after controlling for knowledge in the regressions of attitude and practice, the newspaper dummy variable became insignificant, suggesting that having newspapers as the main media exposure is positively associated with knowledge level. Among the household heads' occupational dummies, only the service dummy variable was found to be significant in the regressions of practice (Model 3 and Model 5). Additionally, as the knowledge level was positive and significant in Model 4, it can be inferred that a higher knowledge level is associated with a higher attitude score. Similarly, the results of Model 5 also indicated that both knowledge and attitude had positive effects on practice, suggesting that higher knowledge and attitude levels result in higher practice scores.

## 6 Discussion

This study aimed to investigate the knowledge, attitudes, and practices (KAP) of the general public in Bangladesh regarding antibiotics use and AMR. The findings revealed diverse background characteristics of the respondents, including age, gender, income, education, marital status, residence, and occupation. These characteristics provide a comprehensive representation of the population under study. Comparing the findings of this study with previous research conducted on KAP regarding antibiotics use and AMR, several similarities and differences emerge. In terms of knowledge, the majority of respondents in our study demonstrated average knowledge levels, which is consistent with similar studies conducted in both Italy and Ethiopia [31,32]. However, our study identified some gaps in knowledge, such as incorrect beliefs about antibiotic resistance stemming from misuse and lack of awareness about the ineffectiveness of antibiotics against viral infections. These findings align with previous research highlighting the persistence of misconceptions and inadequate knowledge among the public [33,34]. Therefore, interventions aiming to improve knowledge should address these specific areas of misconception and provide accurate information about the appropriate use of antibiotics.

Regarding attitudes, our study found that most respondents demonstrated a moderate attitude toward antibiotic use and AMR. This finding is consistent with previous studies conducted in Pakistan, and Australia and Sri Lanka that reported mixed attitudes among the public [35,36]. However, our study identified misconceptions regarding the role of antibiotics in hastening the recovery process after fever and the practice of seeking treatment from multiple doctors if antibiotics are not initially prescribed. These findings highlight the importance of targeted interventions to address these misconceptions and promote appropriate attitudes toward antibiotic use. Efforts should focus on enhancing public awareness about the limitations of antibiotics and the potential consequences of inappropriate use. In terms of practices, our study revealed a significant proportion of respondents exhibiting good practices regarding antibiotic use. This is an encouraging finding, as it indicates a positive trend toward responsible antibiotic use among the public. However, there were instances of self-medication without a doctor's advice, particularly for certain diseases such as ulcers and heart disease. Similar findings have been reported in previous studies, emphasizing the need for interventions targeting specific conditions where self-medication is more prevalent [20,37]. Furthermore, a considerable number of respondents did not understand the importance of completing a full antibiotic course, even if symptoms improved. This highlights the need for educational campaigns to emphasize the importance of adherence to treatment guidelines and the potential consequences of incomplete courses.

The socio-economic determinants identified in this study align with previous research. Education emerged as a significant factor influencing KAP, consistent with findings from various studies conducted in different settings [[38], [39], [40]]. This highlights the crucial role of education in improving knowledge, attitudes, and practices related to antibiotics use and AMR. Media exposure was also influential, with access to newspapers and social media positively impacting knowledge, attitudes, and practices. These findings support the use of diverse media platforms for effective dissemination of information and educational campaigns targeting the public. Therefore, addressing the gaps in public KAP regarding antibiotics use and AMR is crucial for effective antimicrobial stewardship and combating the global threat of antibiotic resistance. The findings of this study highlight the need for targeted educational interventions, the correction of misconceptions, and the promotion of responsible antibiotic use. By enhancing public knowledge, promoting positive attitudes, and encouraging appropriate practices, we can contribute to the preservation of antibiotic effectiveness and the long-term control of AMR.

## 7 Conclusion

The study indicates significant gaps and misconceptions regarding antibiotics use and antimicrobial resistance (AMR) in Bangladesh. Public education campaigns and targeted interventions are essential to enhance knowledge, promote responsible antibiotic use, and combat the growing threat of AMR. These campaigns can utilize various media channels, particularly social media, and newspapers, which were identified as effective awareness building platform for promoting positive KAP regarding antibiotics use and AMR. Further research is needed to assess the long-term impact of such interventions and to develop comprehensive strategies to address AMR at the national level. Policies should also focus on regulating antibiotic sales to prevent over-the-counter availability, enforcing prescription requirements, and promoting appropriate antibiotic use in clinical and community settings.

### 7.1 Limitations

While this study provides valuable insights into the KAP regarding antibiotics use and AMR in Bangladesh, several limitations should be acknowledged. Firstly, the study used a cross-sectional design, restricting causal inferences and long-term trends. The sample may not fully represent the entire population, as it predominantly includes urban residents and individuals with higher education levels. Future research should employ longitudinal designs, objective measures, and more representative sampling techniques to validate and expand upon these findings. Secondly, the study relied on a convenience sampling method, which may introduce selection bias and limit the generalizability of the findings. Future studies should aim for more representative samples to enhance the external validity of the results. Additionally, self-reported measures were used to assess practices subject to social desirability and recall biases. Future research could incorporate objective measures or observational data to validate self-reported practices.

## Funding

This study was not funded by any grant or funding agency.

## Ethics statement

The study received ethical approval from the Institutional Review Board (IRB) of the 10.13039/501100020268Institute of Health Economics (IHE), 10.13039/501100006523University of Dhaka. The statement indicating approval of this research is as follows- “The Institutional Review Board of the 10.13039/501100020268Institute of Health Economics (IHE-IRB), which is approved by the U.S. 10.13039/100000016Department of Health and Human Services Federalwide Assurance (FWA), No. FWA00026031 had reviewed our submissions, both the initial and subsequent responding comments of the IHE-IRB for ethical approval of the proposal titled Public Knowledge, Attitudes, and Practices (KAP) Regarding Antibiotics Use and Antimicrobial Resistance (AMR) in Bangladesh"'. IHE-IRB is providing ethical approval of the proposal."

The ethics approval number is IHEirb562022.

All the respondents were fully informed about the procedures and risks involved and asked for their consent to participate in the interview.

## Data availability statement

The data that support the findings of this study are available in the supplementary material/referenced in the article.

## CRediT authorship contribution statement

**Md Ragaul Azim:** Conceptualization, Formal analysis, Methodology, Project administration, Writing – original draft, Writing – review & editing. **K.M Nafiz Ifteakhar:** Formal analysis, Methodology, Writing – review & editing. **Md Mahfujur Rahman:** Data curation, Formal analysis, Investigation, Writing – original draft. **Quazi Nazmus Sakib:** Conceptualization, Data curation, Writing – original draft.

## Declaration of generative AI and AI-assisted technologies in the writing process

During the preparation of this work, the authors used QuillBot and ChatGPT in order to improve language and readability, with caution. After using these tools, the authors reviewed and edited the content as needed and take full responsibility for the content of the publication.

## Declaration of competing interest

The authors declare that they have no known competing financial interests or personal relationships that could have appeared to influence the work reported in this paper.

## References

[1] Lee Ventola C. (2015).

[2] ANTIMICROBIAL RESISTANCE Global Report on Surveillance.

[3] (2018). https://www.oecd-ilibrary.org/social-issues-migration-health/stemming-the-superbug-tide_9789264307599-en.

[4] Murray C.J., Ikuta K.S., Sharara F., Swetschinski L., Robles Aguilar G., Gray A. (2022 Feb 12). Global burden of bacterial antimicrobial resistance in 2019: a systematic analysis. Lancet.

[5] Laxminarayan R., Matsoso P., Pant S., Brower C., Røttingen J.A., Klugman K. (2016).

[6] (2019). Situational Analysis of Antimicrobial Resistance an Update on Two Years of Implementation of National Action Plans.

[7] Hossain Habib Z. (2016-2021).

[8] Bilal H., Khan M.N., Rehman T., Hameed M.F., Yang X. (2021 Dec 1). Antibiotic resistance in Pakistan: a systematic review of past decade. BMC Infect. Dis..

[9] Tracking AMR Country Self-Assessment Survey (TrACSS) (2022).

[10] Tracking AMR Country Self-Assessment Survey (TrACSS) (2022).

[11] Tracking AMR Country Self-Assessment Survey (TrACSS) (2022).

[12] The Burden of Antimicrobial Resistance (AMR) in Pakistan.

[13] (2023). Bracing for Superbugs Strengthening Environmental Action in the One Health Response to Antimicrobial Resistance [Internet].

[14] Unicomb L.E., Nizame F.A., Uddin M.R., Nahar P., Lucas P.J., Khisa N. (2021 Dec 1). Motivating antibiotic stewardship in Bangladesh: identifying audiences and target behaviours using the behaviour change wheel. BMC Publ. Health.

[15] Hoque R., Ahmed S.M., Naher N., Islam M.A., Rousham E.K., Islam B.Z. (2020 Jan 1). Tackling antimicrobial resistance in Bangladesh: a scoping review of policy and practice in human, animal and environment sectors. PLoS One.

[16] Nahar P., Unicomb L., Lucas P.J., Uddin M.R., Islam M.A., Nizame F.A. (2020 Jul 15). What contributes to inappropriate antibiotic dispensing among qualified and unqualified healthcare providers in Bangladesh? A qualitative study. BMC Health Serv. Res..

[17] Matin M.A., Khan W.A., Karim M.M., Ahmed S., John-Langba J., Sankoh O.A. (2020).

[18] (2015). Antibiotic Resistance: Multi-Country Public Awareness Survey.

[19] Biswas M., Roy M.N., Manik M.I.N., Hossain M.S., Tapu S.T.A., Moniruzzaman M. (2014 Aug 14). Self medicated antibiotics in Bangladesh: a cross-sectional health survey conducted in the Rajshahi City. BMC Publ. Health.

[20] Marzan M., Islam D.Z., Lugova H., Krishnapillai A., Haque M., Islam S. (2021). Knowledge, attitudes, and practices of antimicrobial uses and resistance among public university students in Bangladesh. Infect. Drug Resist..

[21] Hassan M.M., Kalam M.A., Alim M.A., Shano S., Nayem M.R.K., Badsha M.R. (2021 Jul 1). Knowledge, attitude, and practices on antimicrobial use and antimicrobial resistance among commercial poultry farmers in Bangladesh. Antibiotics.

[22] Siam M.H.B., Imran A., Limon M.B.H., Zahid M.H., Hossain M.A., Siddique M.A. (2021 Jun 1). Antibiotic abuse: a cross-sectional study on knowledge, attitude, and behavior among the university students in Dhaka, Bangladesh. Electronic Journal of General Medicine.

[23] Chapot L., Sarker M.S., Begum R., Hossain D., Akter R., Hasan M.M. (2021 Mar 1). Knowledge, attitudes and practices regarding antibiotic use and resistance among veterinary students in Bangladesh. Antibiotics.

[24] Kalam M.A., Alim M.A., Shano S., Nayem M.R.K., Badsha M.R., Al Mamun M.A. (2021 Jun 1). Knowledge, attitude, and practices on antimicrobial use and antimicrobial resistance among poultry drug and feed sellers in Bangladesh. Vet Sci.

[25] Zajmi D., Berisha M., Begolli I., Hoxha R., Mehmeti R., Mulliqi-Osmani G. (2017 Jan 1). Public knowledge, attitudes and practices regarding antibiotic use in Kosovo. Pharm. Pract..

[26] Napolitano F., Izzo M.T., Di Giuseppe G., Angelillo I.F. (2013 Dec 23). Public knowledge, attitudes, and experience regarding the use of antibiotics in Italy. PLoS One.

[27] Miyano S., Htoon T.T., Nozaki I., Pe E.H., Tin H.H. (2022 Aug 1). Public knowledge, practices, and awareness of antibiotics and antibiotic resistance in Myanmar: the first national mobile phone panel survey. PLoS One.

[28] Jairoun A., Hassan N., Ali A., Jairoun O., Shahwan M. (2019 May 6). Knowledge, attitude and practice of antibiotic use among university students: a cross sectional study in UAE. BMC Publ. Health.

[29] The fight against Antimicrobial Resistance is closely linked to the Sustainable Development Goals Why pay attention to antimicrobial resistance (AMR)? Why focus on the Sustainable Development Goals?. http://www.euro.who.int.

[30] (2008). Stop TB Partnership (World Health Organization). Advocacy, Communication and Social Mobilization for TB Control : a Guide to Developing Knowledge, Attitude and Practice Surveys.

[31] Scaioli G., Gualano M.R., Gili R., Masucci S., Bert F., Siliquini R. (2015).

[32] Gebeyehu E., Bantie L., Azage M. (2015 Sep 17). Inappropriate use of antibiotics and its associated factors among urban and rural communities of Bahir Dar city administration, northwest Ethiopia. PLoS One.

[33] McKay R., Mah A., Law M.R., McGrail K., Patrick D.M. (2016). Systematic review of factors associated with antibiotic prescribing for respiratory tract infections. Antimicrobial Agents and Chemotherapy. American Society for Microbiology.

[34] Lambert M., Smit C.C.H., De Vos S., Benko R., Llor C., Paget W.J. (2022). A systematic literature review and meta-analysis of community pharmacist-led interventions to optimise the use of antibiotics. British Journal of Clinical Pharmacology. John Wiley and Sons Inc.

[35] Ahmad T., Khan F.U., Ali S., Rahman A.U., Khan S.A. (2022 Feb 1). Assessment of without prescription antibiotic dispensing at community pharmacies in Hazara Division, Pakistan: a simulated client's study. PLoS One.

[36] Sakeena M.H.F., Bennett A.A., Carter S.J., McLachlan A.J. (2019 Mar 1). A comparative study regarding antibiotic consumption and knowledge of antimicrobial resistance among pharmacy students in Australia and Sri Lanka. PLoS One.

[37] Saravanan R., Raveendaran V. (2013). Antimicrobial resistance pattern in a tertiary care hospital: an observational study. J. Basic Clin. Pharm..

[38] Vazquez-Lago J.M., Lopez-Vazquez P., López-Durán A., Taracido-Trunk M., Figueiras A. (2012 Jun). Attitudes of primary care physicians to the prescribing of antibiotics and antimicrobial resistance: a qualitative study from Spain. Fam. Pract..

[39] Arnau-Sánchez J., Jiménez-Guillén C., Alcaraz-Quiñonero M., Vigueras-Abellán J.J., Garnica-Martínez B., Soriano-Ibarra J.F. (2023 Apr 1). Factors influencing inappropriate use of antibiotics in infants under 3 Years of age in primary care: a qualitative study of the paediatricians' perceptions. Antibiotics.

[40] Hassan A., Alnasser A., Al-Tawfiq J.A., Ahmed H., Ahmed A., Mohammed S. (2021). Primary health centers, first health cluster in eastern province, ministry of health. Journal of Public Health Research.

