# Supplementary material for: Public knowledge, attitudes, and practices (KAP) regarding antibiotics use and antimicrobial resistance (AMR) in Bangladesh
Source: Heliyon. 2023 Oct 18;9(10):e21166. doi: 10.1016/j.heliyon.2023.e21166 (PMC10616402; doi:10.1016/j.heliyon.2023.e21166)
Supplement: Multimedia component 1 [file mmc1.docx]

| 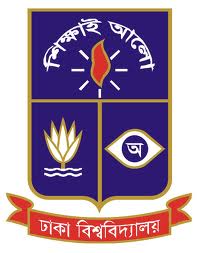 | **Institute of Health Economics**  **University of Dhaka**  Arts Faculty Building (4th Floor)  Dhaka – 1000, Bangladesh  Phone: 9661900-73, Ext.- 8640, 8641  Fax: 88-02-8615583, E-mail: nasrin.sultana@du.ac.bd, Website: www.ihe.ac.bd |  | 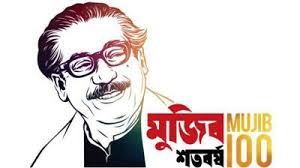 |
| --- | --- | --- | --- |

|  | QID: | \| **0** \| **1** \|  \|  \|  \|  \| \| --- \| --- \| --- \| --- \| --- \| --- \| |
| --- | --- | --- | --- | --- | --- | --- | --- | --- |

**Study title:** Public knowledge, attitudes, and practices (KAP) regarding antibiotics use and antimicrobial resistance (AMR) in Bangladesh

| **Instrument Type :** | **Questionnaire for quantitative survey** |
| --- | --- |
| Respondent : | From different public places (Bus terminal, Market place, Railway Station, Launch terminal and educational institution) |

University of Dhaka

**Consent Form**

Greetings,

My Name is: ---------------------------------------. I have come from the Institute of Health Economics, University of Dhaka. I have come to collect data for the study titled “Public knowledge, attitudes, and practices (KAP) regarding antibiotics use and antimicrobial resistance (AMR) in Bangladesh”. Now, I am explaining the details of the study.

The main objective of the study is to assess the level of knowledge, attitude and, practices (KAP) regarding antibiotics use and antimicrobial resistance (AMR) in Bangladesh.

You have been selected as one of the respected respondents of this study. I would like to ask you some questions regarding this study. Your responses will only be used for research purpose. The name of any respondent of this study will not be mentioned in the report or data set. Nonetheless, there is a possibility of contacting again with the respondent, if needed.

Now I seek your cooperation in this regard. I hope you will response to my questions properly. Your responses may help to design policies as to increase the quality of health care services, thus, the nation and the country will be benefited. Please be assured that if you like to withdraw from the study at any point, you may do so without any restrictions from the investigators.

If you want to know more details of the study, please communicate with Md. Ragaul Azim, Assistant Professor, Institute of Health Economics, University of Dhaka, Chief Investigator of the study, Mobile: 01843-708563. You can ask me if you want to know any further information.

Do you have any further question about this research? **(Advise: If the respondent asks any question, please try to answer properly)**

May I now start to ask you questions in this regard?

Yes --------------1

No ---------------2 (Stop interviewing)

Interviewer’s signature

(Sign after getting proper consent)

|  | Date | \|  \|  \|  \|  \| 2 \| 0 \| 2 \| 2 \| \| --- \| --- \| --- \| --- \| --- \| --- \| --- \| --- \| |
| --- | --- | --- | --- | --- | --- | --- | --- | --- | --- | --- |
|  |  | \| Day \| Month \| Year \| \| --- \| --- \| --- \| |

| Interview starting time | : | \|  \|  \| \| --- \| --- \| | \|  \|  \| \| --- \| --- \| |
| --- | --- | --- | --- | --- | --- | --- | --- |
|  |  | \| Hour \| \| --- \| | \| Minute \| \| --- \| |
| Interview ending time | : | \|  \|  \| \| --- \| --- \| | \|  \|  \| \| --- \| --- \| |
|  |  | \| Hour \| \| --- \| | \| Minute \| \| --- \| |

**Section 1: Socio-demographic Characteristics:**

| Sl. | Question | Response | Code |
| --- | --- | --- | --- |
| 101 | Age of the respondent | \|  \|  \| \| --- \| --- \|   *( Years)* | - |
| 102 | Gender |  | \| Male-------------------------- \| 1 \| \| --- \| --- \| \| Female ---------------------- \| 2 \| |
| 103 | Marital status |  | \| Married--------------------- \| 1 \| \| --- \| --- \| \| Unmarried ----------------- \| 2 \| \| Others (Please specify)--- \| 3 \| |
| 104 | Level of education |  | Passing year (if illiterate, please write ‘0’) |
| 105 | Monthly family income |  | **-** |
| 106 | Place of residence |  | \| Urban------------------------ \| 1 \| \| --- \| --- \| \| Rural ------------------------ \| 2 \| |
| 107 | Occupation |  | \| Business-------------------- \| 1 \| \| --- \| --- \| \| Day labor------------------- \| 2 \| \| Service---------------------- \| 3 \| \| Housewife------------------ \| 4 \| \| Student---------------------- \| 5 \| \| Others (Please specify)--- \| 6 \| |
| 108 | Frequency of visiting healthcare facility in the last one year |  | \| Once------------------------ \| 1 \| \| --- \| --- \| \| Twice----------------------- \| 2 \| \| Three------------------------ \| 3 \| \| Four and above------------ \| 4 \| \| Never------------------------ \| 5 \| |
| 109 | Media exposure |  | \| TV-------------------------- \| 1 \| \| --- \| --- \| \| Newspaper----------------- \| 2 \| \| Social media--------------- \| 3 \| \| Radio------------------------ \| 4 \| \| No media exposure------- \| 5 \| |
| 110 | Level of education of the main earning member of the family |  | Passing year (if illiterate, please write ‘0’) |
| 111 | Occupation of the main earning member of the family |  | \| Business-------------------- \| 1 \| \| --- \| --- \| \| Day labor------------------- \| 2 \| \| Service---------------------- \| 3 \| \| Housewife------------------ \| 4 \| \| Student---------------------- \| 5 \| \| Others (Please specify)--- \| 6 \| |

**Section 2: Knowledge**

| **SL.** | **Question** | **Response** | **Code** |
| --- | --- | --- | --- |
| 201 | Antibiotics are effective for the treatment of bacterial infections |  | 1. Yes  2. No  3. Don’t know |
| 202 | Antibiotics are effective for the treatment of viral infections |  | 1. Yes  2. No  3. Don’t know |
| 203 | Loss of effectiveness of an antibiotic is antibiotic resistance |  | 1. Yes  2. No  3. Don’t know |
| 204 | Antibiotic resistance creases due to missing of an antibiotic dose |  | 1. Yes  2. No  3. Don’t know |
| 205 | The more we use antibiotic, the higher the risk that resistance develops |  | 1. Yes  2. No  3. Don’t know |
| 206 | Antibiotic resistance can develop due to use of antibiotic without doctor’s prescription |  | 1. Yes  2. No  3. Don’t know |
| 207 | After feeling better, one can stop a partially completed antibiotic dose |  | 1. Yes 2. No 3. Don’t know |
| 208 | Antibiotic resistance can develop due to misuse of antibiotic |  | 1. Yes 2. No 3. Don’t know |

**Section 3: Attitudes**

| **SL.** | **Question** | **Response** | **Code** |
| --- | --- | --- | --- |
| 301 | Antibiotics are safe, so they can commonly be used |  | 1. Agree 2. Disagree 3. Neutral |
| 302 | Without prescription, sale of antibiotics should be banned |  | 1. Agree 2. Disagree 3. Neutral |
| 303 | Surplus/ unused antibiotics can be saved for future use or to give someone else |  | 1. Agree 2. Disagree 3. Neutral |
| 304 | Antibiotics speed up the recovery from most coughs and colds |  | 1. Agree 2. Disagree 3. Neutral |
| 305 | It is okay to take antibiotics based on the suggestion of a medicine seller |  | 1. Agree 2. Disagree 3. Neutral |
| 306 | I should take antibiotics to avoid getting a more severe illness, when I suffer from cough and cold |  | 1. Agree 2. Disagree 3. Neutral |
| 307 | Antibiotic speeds up recovery when I get a fever |  | 1. Agree 2. Disagree 3. Neutral |
| 308 | If a doctor does not prescribe an antibiotic when I think one is needed, I will go to another doctor |  | 1. Agree 2. Disagree 3. Neutral |

**Section 4: Practices**

| SL. | Question | Response | Code |
| --- | --- | --- | --- |
| 401 | Before using antibiotics, do you ask a doctor? |  | 1. Yes  2. No |
| 402 | Do you always fulfill the antibiotic course? |  | 1. Yes  2. No |
| 403 | How do you mostly intake antibiotics? |  | 1. Physician’s prescription  2. Self-medication  3. Suggested by friends/relatives  4. Suggested by medicine seller  5. According to previous prescription |
| 404 | After taking some doses of antibiotics, if you feel better, do you still complete the full dose of treatment? |  | 1. Yes  2. No |
| 405 | Have you been sick in the last 12 months? |  | 1. Yes  2. No |
| 406 | If yes, how often? |  |  |
| 407 | What kind of disease did you suffer from? |  | (See code 406) |
| 408 | In the last 12 months, have you taken any antibiotic? |  | 1. Yes  2. No |
| 409 | In the last 12 months, how many times did you use antibiotics? |  | 1. Once  2. Twice  3. More than two times |
| 410 | How did you get the last course of antibiotics that you consumed? |  | 1. Physician’s prescription  2. Self-medication  3. Suggested by friends/relatives  4. Suggested by medicine seller  5. According to previous prescription |
| 411 | What was the reason for last taking antibiotics that you consumed? |  | 1. Cold  2. Fever  3. Bacterial Infection  4. Other |
| 412 | In the last 12 months, did you fulfill the antibiotic course? |  | 1. Yes  2. No |

**Code-406**

| 1. Normal fever, cold, cough  2. Pain in different limbs (hand, leg, abdomen, head, back)  3. Acute fever  4. Diarrhea  5. Skin disease  6. Dental case  7. Influenza  8. Diabetic  10. Anemia | 11. Sinusitis  12. Tuberculosis  13. Eye problem  14. Heart Disease  15. ENT  16. Asthma/ Cold allergy  17. Kidney disease  18. Cold/dust allergy  19. Paralysis  20. Ulcer  21. back pain | 22. Spine problems  23. Mental disease  24. Tumor  25. Rheumatic fever  26. Cancer  27. Arthritis  28. Epilepsy  29. Liver cirrhosis  30. Urine infection  31. Stomach problem  32. Rat kana  33. Sexual disease  34.Piles | 35. Stroke  36. Causality (both accident and fighting )  37. neurosis  38. Jaundice  39. Typhoid  40. Dengue  41. Chikungunya  42. Amshay  43. Gastric  44. Malaria  45. Corona/Covid-19  46. Other (Specify) |
| --- | --- | --- | --- |
